# Supplementary material for: Value representations in the rodent orbitofrontal cortex drive learning, not choice
Source: eLife. 2022 Aug 17;11:e64575. doi: 10.7554/eLife.64575 (PMC9462853; doi:10.7554/eLife.64575)
Supplement: Transparent reporting form [file elife-64575-transrepform1.pdf]

eLIFE
1st Floor
24 Hills Road
Cambridge CB2 1JP, UK
P 01223 855340
W elifesciences.org
T @elife

eLife's transparent reporting form

We encourage authors to provide detailed information *within their submission* to facilitate the interpretation and replication of experiments. Authors can upload supporting documentation to indicate the use of appropriate reporting guidelines for health-related research (see EQUATOR Network), life science research (see the BioSharing Information Resource), or the ARRIVE guidelines for reporting work involving animal research. Where applicable, authors should refer to any relevant reporting standards documents in this form.

If you have any questions, please consult our Journal Policies and/or contact us: editorial@elifesciences.org.

Sample-size estimation

- You should state whether an appropriate sample size was computed when the study was being designed
- You should state the statistical method of sample size computation and any required assumptions
- If no explicit power analysis was used, you should describe how you decided what sample (replicate) size (number) to use

Please outline where this information can be found within the submission (e.g., sections or figure legends), or explain why this information doesn't apply to your submission:

No explicit power analysis was used. We aimed for a sample size consistent with previous studies using similar tools. (Methods, page 13)

Replicates

- You should report how often each experiment was performed
- You should include a definition of biological versus technical replication
- The data obtained should be provided and sufficient information should be provided to indicate the number of independent biological and/or technical replicates
- If you encountered any outliers, you should describe how these were handled
- Criteria for exclusion/inclusion of data should be clearly stated
- High-throughput sequence data should be uploaded before submission, with a private link for reviewers provided (these are available from both GEO and ArrayExpress)

Please outline where this information can be found within the submission (e.g., sections or figure legends), or explain why this information doesn't apply to your submission:

eLife Sciences Publications, Ltd is a limited liability non-profit non-stock corporation incorporated in the State of Delaware, USA, with company number 5030732, and is registered in the UK with company number FC030576 and branch number BR015634 at the address 1st Floor, 24 Hills Road, Cambridge CB2 1JP | August 2014

The electrophysiology experiment measured 477 units from 51 sessions in six rats (Results, page 5; Figure 3 legend). Spikes were detected and assigned to units using an amplitude threshold and manual cluster cutting. No cut units were excluded. (Methods, page 15). We observed that a small fraction of trials showed apparent artifacts in which some units appeared to have extremely high firing rates (we suspect this was due to motion of the implant or tether). We excluded these units for these trials based on Median Absolute Deviation (Leys, et al., 2013) with a conservative threshold of 3. (Methods, page 15)

The optogenetics experiment measured 577 sessions in nine rats (Results, page 8; Figure 4 legend). The sham optogenetics experiment measured 109 sessions in four rats (Results, page 8; Figure 4 legend). No sessions were excluded. We set an upper limit of 15 seconds on the length of illumination of the brain – on trials where a scheduled illumination would have lasted longer than this, the inactivation was terminated and the trial excluded from analysis. (Methods, page 16)

Statistical reporting

- Statistical analysis methods should be described and justified
- Raw data should be presented in figures whenever informative to do so (typically when N per group is less than 10)
- For each experiment, you should identify the statistical tests used, exact values of N, definitions of center, methods of multiple test correction, and dispersion and precision measures (e.g., mean, median, SD, SEM, confidence intervals; and, for the major substantive results, a measure of effect size (e.g., Pearson's r, Cohen's d)
- Report exact p-values wherever possible alongside the summary statistics and 95% confidence intervals. These should be reported for all key questions and not only when the p-value is less than 0.05.

Please outline where this information can be found within the submission (e.g., sections or figure legends), or explain why this information doesn't apply to your submission:

Exact p-values and measures of effect size can be found in the Results section of the main text.

Details of statistical methods can be found in the Methods section.

(For large datasets, or papers with a very large number of statistical tests, you may upload a single table file with tests, Ns, etc., with reference to sections in the manuscript.)

Group allocation

- Indicate how samples were allocated into experimental groups (in the case of clinical studies, please specify allocation to treatment method); if randomization was used, please also state if restricted randomization was applied
- Indicate if masking was used during group allocation, data collection and/or data analysis

Please outline where this information can be found within the submission (e.g., sections or figure legends), or explain why this information doesn't apply to your submission:

The randomization critical for our experiments derives from the probabilistic structure of the task (probabilistic rewards, probabilistic block flips, probabilistic inhibition).

The electrophysiology, optogenetics, and sham optogenetics experiments were run at different times, and randomization was not used to assign rats to experiments.

The optogenetics and sham optogenetics rats were placed into and removed from the behavioral chamber by technicians blind to the experiment being run.

Additional data files ("source data")

- We encourage you to upload relevant additional data files, such as numerical data that are represented as a graph in a figure, or as a summary table
- Where provided, these should be in the most useful format, and they can be uploaded as "Source data" files linked to a main figure or table
- Include model definition files including the full list of parameters used
- Include code used for data analysis (e.g., R, MatLab)
- Avoid stating that data files are "available upon request"

Please indicate the figures or tables for which source data files have been provided:

Processed data and analysis code will be made available on Dryad upon publication

eLife Sciences Publications, Ltd is a limited liability non-profit non-stock corporation incorporated in the State of Delaware, USA, with company number 5030732, and is registered in the UK with company number FC030576 and branch number BR015634 at the address 1st Floor, 24 Hills Road, Cambridge CB2 1JP | August 2014
